# Polarization-entangled photon generation using partial spatially coherent pump beam

**DOI:** 10.1038/s41598-017-12376-6

**Published:** 2017-09-21

**Authors:** Yaseera Ismail, Stuti Joshi, Francesco Petruccione

**Affiliations:** 1 University of KwaZulu-Natal, School of Chemistry and Physics, Quantum Research Group, Durban, 4000 South Africa; 2 National Institute for Theoretical Physics (NITheP), KwaZulu-Natal, South Africa

## Abstract

The generation of two photon fields, to date, has been demonstrated utilizing a fully coherent pump beam. In this paper we demonstrate, the theoretical and experimental generation of polarization entangled single photon pairs by varying the spatial coherence of the pump beam. The effect of the pump beam spatial coherence on the visibility of a polarization-entangled single photon source is investigated. A comparison of the visibility measurements using a fully coherent and partially coherent pump beam is performed. It is shown that the partial coherence of the pump beam contributes to an increase in the visibility. The coherence properties of the beam are significant for free-space optical transmission in particular for long range free-space quantum communication.

## Introduction

The process of spontaneous parametric down-conversion (SPDC)^1^ is the foremost popular method of producing entangled photons and has acquired immense attention of late due to its potential applications in quantum communication especially for long range free-space and satellite quantum key distribution^2–4^. Governed by the laws of conservation of energy and momentum, high frequency pump photons split into highly correlated low frequency photon pairs whereby the generated photon pairs form an entangled twin photon state^1,5^. Photon pairs can be entangled in time and energy, phase and momentum and angular position and orbital angular momentum depending on the properties of the crystal and the pump beam. The aforementioned entanglement properties associated with the fourth-order correlation have been demonstrated in previous studies^6–11^.

For many quantum applications, the two-photon interference effect corresponding to temporal^12,8^, spatial^13,14^, and angular interference^15,16^ has been demonstrated. Two-photon fields entangled in different degrees of freedom can provide additional resources to prepare the entangled quantum states for quantum communication.

The coherence properties of the entangled photon fields are affected by the pump properties and crystal parameters^5,17–22^. In particular, the transfer of angular momentum of the pump beam^23^ and the effect of the pump transverse width^19^ on the down-converted photons have been investigated both theoretically and experimentally. Thus far, entangled photons are experimentally generated by considering the pump beam to be fully spatially coherent (FSC). However, in many practical applications partially spatially coherent light demonstrated robustness over fully coherent light^24^. Of late, there have been reports of theoretical studies focusing on integrating partial spatial coherence to an entangled photon field and studying the connection between the partial coherence and entanglement. For instance, the mathematical investigation of the duality between partial coherence and entanglement describes the propagation, diffraction and interference of bi-photon fields in optical linear systems^25^. In particular, the beam properties such as spectral intensity and state of polarization are intrinsically connected to the spatial coherence of the pump beam^26^.

The generation of the biphoton field is not limited to SPDC, it was recently shown that the process of four wave mixing within a hot Rubidium atomic vapor cell, resulted in the production of bright narrowband biphotons where a violation of the Cauchy-Schwartz inequality was demonstrated^27^. It was also shown that within an atomic medium it is possible to study the effects of the temporal correlation by producing the narrowband biphoton field using frequency-bin entanglement^28^.

In the present paper, applying the process of SPDC, we demonstrate a novel pump source for entangled photons by incorporating a scheme to produce partial spatial coherence of the pump beam. In doing so we achieve a varying partial spatial coherent pump photon source. We show for the first time the partially spatially coherent source for a quantum system. An experimental study is performed to investigate the effects of the pump spatial coherence, on the properties of the polarization-entangled photon field. We observe the non-classical behaviour of the two-photon field for different values of the pump spatial coherence lengths by testing the visibility and the Clauser, Horne, Shimony and Holt (CHSH) inequality^29^. Furthermore, the associated measurements were performed on the conventional coherent pump source (FSC) and compared with the partially spatially coherent pump beam. Since the state of polarization and coherence are intimately connected^30^, it can be expected that the polarization properties are influenced by the coherence. The present study is significant for potential applications in quantum information processing for free-space quantum communication.

## Theoretical Background

**Figure 1:**
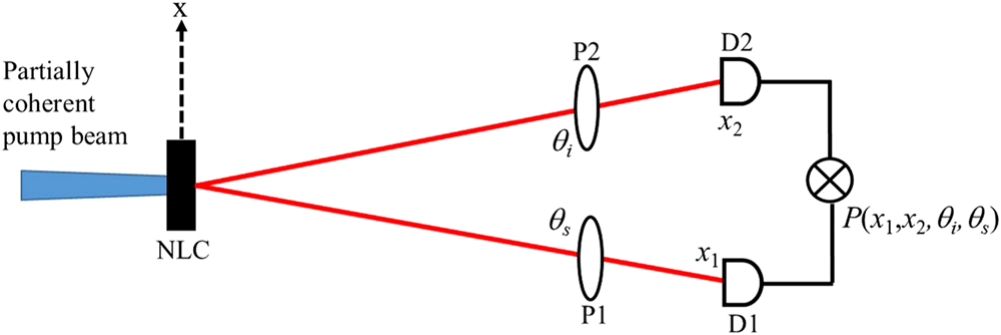
Schematic setup used to study the influence of the pump spatial coherence on the polarization entangled photons.

Throughout the paper, subscripts *p*, *s*, and *i* stand for pump, signal, and idler respectively. The two-photon state $|{\rm{\Psi }}\rangle$ generated by a partial spatial coherent pump source, as shown in Fig. 1, is expressed as^26^,

1 $$|{\rm{\Psi }}\rangle =A\exp (i{\rm{\Delta }}kL\mathrm{/2)}\iint d{q}_{s}d{q}_{i}{V}_{p}({q}_{p})\exp (i{\rm{\Delta }}qL\mathrm{/2)}{\rm{sinc}}({\rm{\Delta }}qL\mathrm{/2)}{\hat{b}}_{s}^{\dagger }({q}_{s}){\hat{b}}_{i}^{\dagger }({q}_{i})|\mathrm{0,}\,0\rangle ,$$

where $|0,\,0\rangle$ is the vacuum state, *L* is the length of the nonlinear crystal and *V*_*p*_(*q*_*p*_) is the strength of the pump field with *q*_*p*_ = *q*_*s*_ + *q*_*i*_ is the transverse coordinate of the wave-vector **k**_*p*_ of the pump beam. For the paraxial approximation Δ*k* and Δ*q* are given by,

2a $${\rm{\Delta }}k={k}_{p}-{k}_{s}-{k}_{i},$$

2b $${\rm{\Delta }}q=\frac{{q}_{p}^{2}}{2{k}_{p}}-\frac{{q}_{s}^{2}}{2{k}_{s}}-\frac{{q}_{i}^{2}}{2{k}_{i}},$$

where *k*_*p*_, *k*_*s*_ and *k*_*i*_ are the wavenumber of the pump, the signal and the idler respectively. For Δ*q* < 0, sinc(Δ*qL*/2) = exp$(-\gamma {\sqrt{\Delta q}}^{2}L/2)$ and *γ* = 0.455^31^.

For the theoretical calculation we considered partially coherent pump beam of Gaussian Schell-Model type. The correlation function for the Gaussian Schell-Model pump beam field is given by^32^,

3 $$\langle V({\rho }_{1}){V}^{\ast }({\rho }_{2})\rangle ={A}_{p}^{2}\exp (-\frac{{\rho }_{1}^{2}+{\rho }_{2}^{2}}{2{\sigma }^{2}}-\frac{{({\rho }_{2}-{\rho }_{1})}^{2}}{4{\delta }^{2}}),$$

where *ρ*_1_ and *ρ*_2_ are two transverse points in the source plane.

The probability of coincidence of the signal at *x*_1_ and the idler at *x*_2_ is given by^25^,

4 $$P({x}_{1},{x}_{2},{\theta }_{s},{\theta }_{i})=|{\rm{\Psi }}\rangle {E}_{s}^{-}({x}_{1},{\theta }_{s}){E}_{i}^{-}({x}_{2},{\theta }_{i}){E}_{i}^{+}({x}_{2},{\theta }_{i}){E}_{s}^{+}({x}_{1},{\theta }_{s})|{\rm{\Psi }}\rangle ,$$

where ${E}_{s}^{+}({x}_{1},{\theta }_{s})$ and ${E}_{i}^{+}({x}_{2},{\theta }_{i})$ denote the positive frequency parts of the electric fields of the signal and the idler arriving at detectors D1 and D2 respectively. Similarly, ${E}_{s}^{-}({x}_{1},{\theta }_{s})$ and ${E}_{s}^{-}({x}_{2},{\theta }_{i})$ represent the negative frequency parts, *θ*_*s*_ and *θ*_*i*_ are the polarization angles of polarizers P1 and P2 inserted in the path of the signal and the idler field respectively. The positive and negative frequency parts of the signal and the idler at the detection plane can be expressed as^25^,

5a $${E}_{j}^{+}(x,{\theta }_{j})=\,\cos ({\theta }_{j})\int d{q}_{j}{H}_{j}(x,{q}_{j}){\hat{b}}_{j}({q}_{j}),$$

5b $${E}_{j}^{-}(x,{\theta }_{j})=\,\cos ({\theta }_{j})\int d{q}_{j}{H}_{j}^{\ast }(x,{q}_{j}){\hat{b}}_{j}^{\dagger }({q}_{j}),$$

where ${\hat{b}}_{j}({q}_{j})$ and ${\hat{b}}_{j}^{\dagger }({q}_{j})$ are the annihilation and creation operator respectively and *j* = *s*, *i*. Here, *H*_*j*_(*x*, *q*_*j*_) is the response of the signal (idler) system in free-space.

Substituting Equations (1), (2) and (5) into Equation (4) we obtained an expression for probability of coincidence detection of signal and idler in terms of pump cross-spectral density function as given by,

6 $$\begin{array}{c}P({x}_{1},{x}_{2},{\theta }_{s},{\theta }_{i})=|A{|}^{2}{\cos }^{2}({\theta }_{s}){\cos }^{2}({\theta }_{i})\int \int \int \int \,d{\rho }_{s}d{\rho }_{s}^{^{\prime} }d{\rho }_{i}d{\rho }_{i}^{^{\prime} }W({\rho }_{s},{\rho }_{s}^{^{\prime} },{\rho }_{i},{\rho }_{i}^{^{\prime} })\\ \quad \quad \quad \quad \quad \quad \quad \times \langle {h}_{s}({x}_{1},{\rho }_{s}){h}_{s}^{\ast }({x}_{1},{\rho }_{s}^{^{\prime} }){h}_{i}({x}_{2},{\rho }_{i}){h}_{i}^{\ast }({x}_{2},{\rho }_{i}^{^{\prime} })\rangle ,\end{array}$$

where, 〈….〉 is the ensemble average over all the realizations of the transverse coordinates, *h*_*j*_(*x*, *ρ*_*j*_) is the Fourier transform of *H*_*j*_(*x*, *q*_*j*_) with respect to *ρ*_*j*_, and *W*(*ρ*_*s*_, *ρ*′_*s*_, *ρ*_*i*_, *ρ*′_*i*_) is the cross-spectral density function of the partially coherent pump field which is the function of the ensemble average of the pump field over different realizations. The Fourier transform function *h*_*j*_(*x*, *ρ*_*j*_) of *H*_*j*_(*x*, *q*_*j*_) is given by,

7 $${h}_{j}(x,{\rho }_{j})=\sqrt{-\frac{i{k}_{j}}{2\pi z}}\exp (\frac{i{k}_{j}}{2z}{(x-{\rho }_{j})}^{2})\mathrm{.}$$

The cross-spectral density function *W*(*ρ*_*s*_, *ρ*′_*s*_, *ρ*_*i*_, *ρ*′_*i*_) is given by,

8 $$\begin{array}{rcl}W({\rho }_{s},{\rho }_{s}^{^{\prime} },{\rho }_{i},{\rho }_{i}^{^{\prime} }) & = & \frac{4\pi {k}_{p}}{L\sqrt{{\gamma }^{2}+1}}\langle {V}_{p}(-\frac{{\rho }_{s}+{\rho }_{i}}{2}){V}_{p}^{\ast }(-\frac{{\rho }_{s}^{^{\prime} }+{\rho }_{i}^{^{\prime} }}{2})\rangle \\  &  & \times \exp (-b{({\rho }_{s}^{^{\prime} }-{\rho }_{i}^{^{\prime} })}^{2}-c({\rho }_{s}-{{\rho }_{i}}^{2})),\end{array}$$

where,

9a $$b=\frac{{k}_{p}}{4L(-i+\gamma )},$$

and

9b $$c=\frac{{k}_{p}}{4L(i+\gamma )}\mathrm{.}$$

Here $\langle {V}_{p}(-\frac{{\rho }_{s}+{\rho }_{i}}{2}){V}_{p}^{\ast }(-\frac{{\rho }_{s}^{^{\prime} }+{\rho }_{i}^{^{\prime} }}{2})\rangle$ represents the correlation function of the partially coherent pump beam and can be calculated using Equation (3) as,

10 $$\begin{array}{rcl}\langle {V}_{p}(-\frac{{\rho }_{s}+{\rho }_{i}}{2}){V}_{p}^{\ast }(-\frac{{\rho }_{s}^{^{\prime} }+{\rho }_{i}^{^{\prime} }}{2})\rangle  & = & {A}_{p}^{2}\exp (-\eta {({\rho }_{s}+{\rho }_{i})}^{2}\\  &  & -\eta {({\rho }_{s}^{^{\prime} }+{\rho }_{i}^{^{\prime} })}^{2}+\eta ^{\prime} ({\rho }_{s}+{\rho }_{i})({\rho }_{s}^{^{\prime} }+{\rho }_{i}^{^{\prime} })),\end{array}$$

where,

11a $$\eta =\frac{1}{16{\delta }^{2}}+\frac{1}{8{\sigma }^{2}},$$

11b $$\eta ^{\prime} =\frac{1}{8{\delta }^{2}},$$

${A}_{p}^{2}$ is a constant, *σ* and *δ* represents the beam waist and the pump coherence length respectively. Substituting Equations (7), (8) and (10) into Equation (6) and after some calculation the probability of coincidence detection can be expressed as,

12 $$\begin{array}{c}P({x}_{1},{x}_{2},{\theta }_{s},{\theta }_{i})=\frac{C{\cos }^{2}({\theta }_{s}){\cos }^{2}({\theta }_{i})}{\sqrt{(2{A}_{1}{(-a+c+\eta )}^{2}{(a-2c)}^{2}(a-2\eta )({A}_{2}+{A}_{3}-2{a}^{2}{x}_{2}))}}\\ \quad \quad \quad \quad \quad \quad \quad \times \exp (\frac{{a}^{2}{x}_{1}^{2}}{-a+c+\eta })\exp (\frac{{a}^{2}{({x}_{1}\mathrm{(2}a-4\eta +\eta ^{\prime} )+{x}_{2}\eta ^{\prime} )}^{2}}{2{A}_{1}(a-2\eta )})\\ \quad \quad \quad \quad \quad \quad \quad \times \exp (\frac{4{a}^{2}{(-a{x}_{2}+c({x}_{1}+{x}_{2})+({x}_{2}-{x}_{1})\eta )}^{2}}{4{A}_{1}(a-2c)({A}_{3}-2{a}^{2}{x}_{2}+{A}_{2})})\\ \quad \quad \quad \quad \quad \quad \quad \times \exp (\frac{{(a\eta ^{\prime} {A}_{1}({x}_{1}-{x}_{2})-2a(b-\eta )(a-2c)({x}_{1}\mathrm{(2}a-4\eta +\eta ^{\prime} )+{x}_{2}\eta ^{\prime} ))}^{2}}{4{A}_{1}(a-2c)({A}_{3}-2{a}^{2}{x}_{2}+{A}_{2})}),\end{array}$$

where *a* = *ik*_*p*_/4*z* and *A*_1_, *A*_2_, *A*_3_ can be expressed in terms of *a*, *b*, *c*, *η*, *η*′.

13a $${A}_{1}=2{a}^{2}+2a(b-\eta )-4b\eta -4{\eta }^{2}+{\eta ^{\prime} }^{2},$$

13b $${A}_{2}=\frac{2{a}^{2}+2ab-4ac-4bc+2a\eta -4c\eta +{\eta ^{\prime} }^{2}}{\mathrm{2(}a-2c)},$$

13c $${A}_{3}=-\frac{\mathrm{2(}a-2\eta )(b-\eta {)}^{2}}{{A}_{1}}\mathrm{.}$$

Equation (12) shows the probability of coincidence detection for polarization entangled photons as a function of different parameters. The parameter *C* is a function of *k*, *z* and *γ*, which were kept constant during the experiment. The wavelength of the pump beam *λ* = 405 nm, length of the nonlinear crystal *L* = 7 mm and the distance between nonlinear crystal and detectors *z* = 500 mm. The visibilities of the polarization entangled photons for different values of the pump coherence lengths were calculated by fixing the aforementioned parameters using Equation (12). The coincidence counts were determined under the condition that both the detectors (D1 and D2) are fixed at *x*_1_ = *x*_2_ = 0. For the visibility measurements, polarizer, P1, is at *θ*_1_ = 0° and 45° (rectilinear and diagonal basis respectively) while the polarizer, P2, is rotated from *θ*_2_ = 0 to 2*π*.

## Experimental Setup

**Figure 2:**
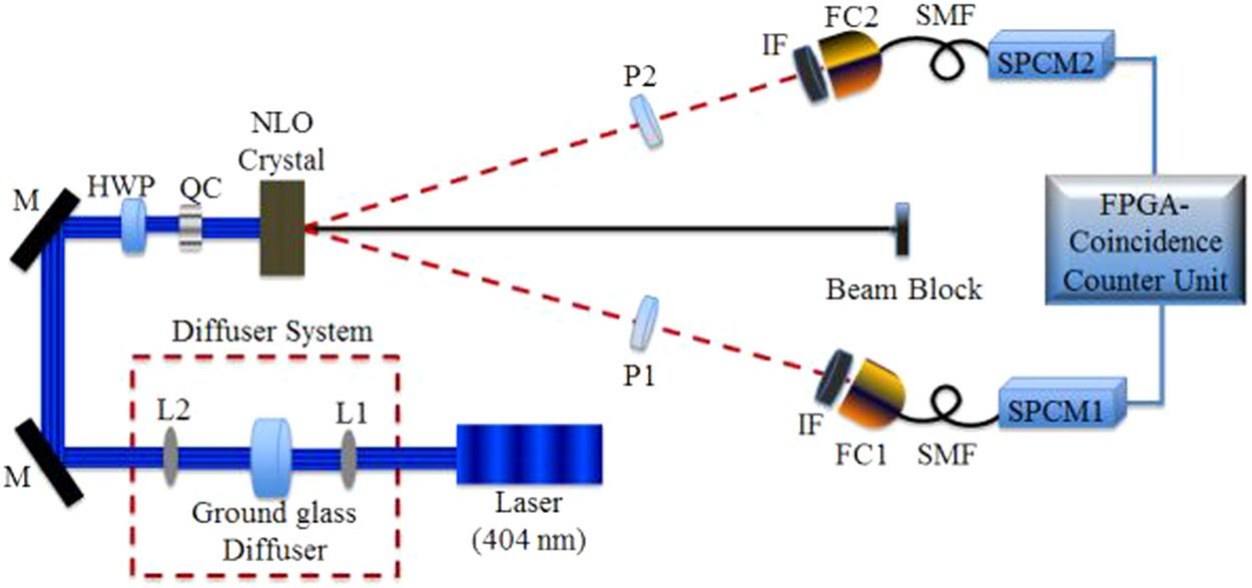
Schematic of an entangled photon source comprising of a pump laser lasing as 405 nm, diffuser system containing a ground glass diffuser and lens system, mirrors (M), a Half Wave Plate (HWP), a Quartz Crystal (QC), BBO crystal, a polarizer in each arm followed by infra-red filters (IF) and a fiber coupler connected to single photon detectors (SPCM1 and SPCM2) via polarization maintaining fibers to the in-house coincidence counting unit (CCU) comprising of a Field Programmable Gated Array (FPGA).

To reproduce the entanglement scheme incorporating the novelty of the partial spatial coherent pump, we implement the process of SPDC utilizing a nonlinear crystal in a type-I concatenated configuration as presented in Fig. 2. The partial spatial coherence applied to the quantum system was investigated by means of performing measurements in the polarization degree of freedom.

The partially spatially coherent (PSC) pump beam was implemented by propagating the initial pump beam (405 nm) through a rotating ground glass diffuser (Fig. 2). A collection lens (L2 = 50 mm) was used to collimate the pump beam coming out of the diffuser. The spatial coherence length was varied by varying the size of the beam incident on the diffuser whereby the coherence length was calculated by, $\delta =\frac{3.832{\lambda }f}{2\pi d}$ ^33^, where *λ* is the wavelength of the pump beam, *f* is the focal length of lens *L*_2_ and *d* is the diameter of the beam imposed onto the ground glass diffuser. Non-collinear photon pairs at a down-converted wavelength of 810 nm were produced by two type-I Beta Barium Borate (BBO) crystals (7 × 7 × 0.7 mm) concatenated using a partially coherent pump beam laser lasing at 405 nm. A quartz crystal (QC) was used to remove unwanted phase shifts. The polarization of the pump beam was controlled using a half wave plate (HWP) which was set to diagonally polarized light relative to the BBO axes allowing for an equal probability of horizontal and vertical polarized photons being generated (signal and idler) in accordance to the state

14 $$|{\rm{\Psi }}\rangle =\frac{1}{\sqrt{2}}[|{V}_{s}\rangle |{V}_{i}\rangle +\exp (-i\varphi )|{H}_{s}\rangle |{H}_{i}\rangle ],$$

where, $|{V}\rangle$ and $|{H}\rangle$ are the vertical and horizontal states respectively and *ϕ* is the phase difference.Since the source was built to perform measurements in the polarization degree of freedom, a linear polarizer was placed in each of the free arms (signal and idler) for measurement purposes. The single photons were collected using free-space to fiber couplers containing a collecting lens with a focal length of 11 mm. Single mode fibers (SMF) were used to couple from the fiber couplers to the single photon avalanche detectors/modules (SPCM) with efficiencies of 65% and dark count rates of the order 100/sec. Coincidence detection was acquired using a multi-channel Coincidence-Counting Unit (CCU) comprising of integrated circuit components. The signals from the SPCM registered as inputs in the CCU, which were a combination of random 2-fold coincidences. The pulses registered as two outputs into a field programmable gate array (FPGA). Coincidence counts were obtained for integration time intervals ranging from 1 s to 5 s for better accuracy within a time window of 7 ns

## Results and Discussion

The coherence length was varied for different magnitudes of partial spatial coherence and determined by measuring the beam diameter at the plane of the ground glass diffuser. Coherence lengths ranging from 27 *μ*m to 112 *μ*m were achieved.

To verify that our partially spatially coherent source was producing entangled photons, we first and foremost analyzed the correlation fringes for the rectilinear and diagonal basis by setting the linear polarizer P1, *α* to 0° and 45° respectively while varying the orientation of the second polarizer P2, *β*:0° to 360°.

**Figure 3:**
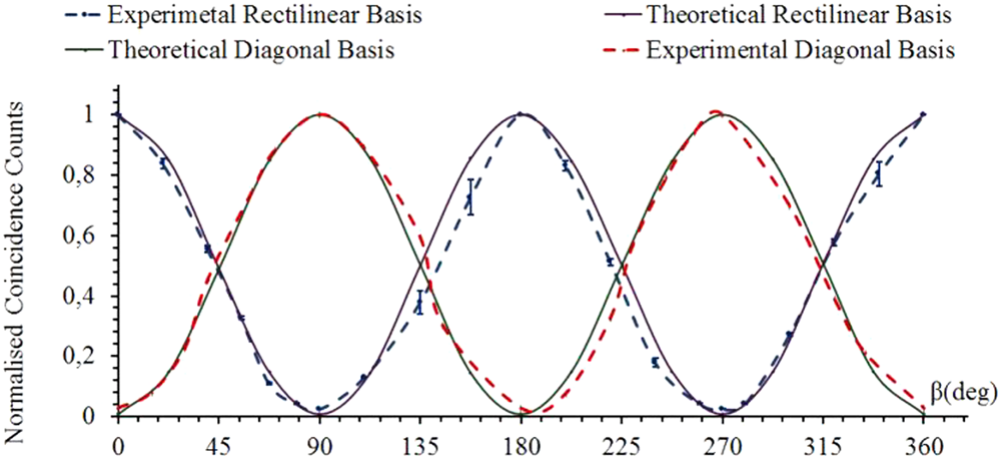
Correlation fringes for the rectilinear (*α* = 0°) and diagonal basis (*α* = 45°) while varying the orientation of the second polarizer (*β* :0° to 360°) for the pump beam coherence length of 112 *μ*m.

The normalized coincidence counts obtained from the experimental data is plotted in Fig. 3 for the pump coherence length of 112 *μ*m. As expected the coincidence counts showed a sinusoidal behavior. A high fringe visibility of 95.1 ± 3.4% and 94.7 ± 3.9% was obtained for the rectilinear (dashed blue curve) and diagonal basis (dashed red curve) respectively. The corresponding theoretical visibility fringes for the rectilinear and the diagonal basis using Equation (12) are plotted by the purple and green solid curves respectively. The experimental visibility curves follow the same trends as the theoretical curves (as per Equation (12)). However, there is a slight shift in the position of maximum and minimum coincidence counts which is due to slight misalignment in the experimental setup. To characterize the source robustness, the visibility of the polarization photons were measured for different values of the pump beam coherence lengths. The results of the experimental visibilities for the FSC and varying coherence lengths of PSC are presented in Table 1, including the corresponding theoretically calculated visibility attained from Equation (12). From these results, it was observed that the visibility of the coincidence fringes increases as the spatial coherence length of the pump beam decreases. The coherence lengths were varied by varying the pump beam size at the plane of the ground glass diffuser. As the beam size decreases the wave-function becomes more separable resulting in an increase of the field coherence length^25^. Due to this the field becomes less entangled and therefore the visibility is lower as proposed theoretically by Saleh *et al*.^25^. The decrease in the visibility of the biphoton field with respect to varying pump coherence length is shown in Table 1. Our results agree with the theoretical studies based on the aforementioned paper^25^ and relate the fringe visibility of the entangled photons to the spatial coherence of the pump beam. For the coherence length of 112 *μ*m (higher coherence, smaller beam size) the visibility obtained is less than that obtained for 27 *μ*m (less coherence, larger beam size). The theoretical model predicts a small change in the visibility with the variation of pump spatial coherence length. The visibility measurements obtained are also influenced by the pump beam waist and the pump coherence length.

**Table 1:** Visibility obtained from the correlation fringes for the rectilinear (*α* = 0°) and diagonal basis (*α* = 45°) for a FSC beam and varying coherence length of the PSC beam.

Pump Beam Size	Pump Beam Coherent Length	Rectilinear Basis	Diagonal Basis	Theoretical Visibility
—	FSC	90.1 ± 1.3 %	90.6 ± 1.3 %	97%
110 *μ*m	112 *μ*m	95.1 ± 3.4 %	94.7 ± 3.9 %	98.5%
350.5 *μ*m	35 *μ*m	95.8 ± 2.9 %	95.6 ± 3.1 %	98.7%
456.8 *μ*m	27 *μ*m	96.9 ± 3.1 %	97.1 ± 2.8 %	98.9%

The aforementioned parameters were determined experimentally and adopted into the theoretical model for both cases, PSC and FSC.

The verification of entanglement lies in the violation of the CHSH inequality performed by determining the expected correlation of entanglement. Therefore a measurement of CHSH was performed for different values of the pump coherence lengths. The coincidence counts were recorded for 5 s. The following set of optimum polarization orientations were chosen for the linear polarizer *α* = 0°, *α*′ = 45°, *β* = 22.5° and *β*′ = 67.5°. Four sets of experimental runs were conducted corresponding to the four sets of expectation values in the definition of the CHSH inequality^29^ and the overall results are presented in Table 2 for the fully and partially spatially coherent pump beam.

**Table 2:** Measured violation of the CHSH inequality for the fully (FSC) and varying values of coherence length of the partially spatially coherent (PSC) pump beam.

Pump Beam Coherent Length	CHSH Inequality	Percentage(%)
FSC	2.70	2.0
112 *μ*m	2.76	0.2
35 *μ*m	2.77	0.2
27 *μ*m	2.78	0.2

These results show that for the coherence length of 112 *μ*m, the CHSH inequality parameter obtained was *S* = 2.76 with a percentage error of 0.2 which according to the local realism theory is |*S*| < 2 and 2 < |*S*| ≤ 2$\sqrt{2}$ according to theoretical limit of quantum mechanics. As performed previously, the robustness of the system was determined in terms of the CHSH inequality for various values of the pump beam coherence lengths. The results obtained in Table 2 show that the degree of entanglement increases with the decrease of the pump spatial coherence. Compared to alternative methods of producing the biphoton field such as frequency-bin entanglement within an atomic medium^28^, the SPDC source presented here, has a stronger violation of the CHSH Inequality. It is worth noting however that within the atomic medium it is possible to produce triplet fields by the process of parametric amplification six-wave mixing. Although these fields are weaker than the biphoton field, it provides opportunities to engineer three-channel entangled imaging on a chip^34^.

## Conclusion

The effects of spatial coherence of the pump beam on an entangled single photon source was studied. The PSC pump beam measured a higher visibility and CHSH inequality in comparison to the FSC pump beam. The experimental results showed that it is possible to obtain a tunable polarization entangled photon source by tailoring the pump beam coherence length. Of late, it has been shown theoretically that the detection probability of the two-photon field generated by the partially spatially coherent pump beam is higher and less susceptible to atmospheric turbulence than the two-photon field produced by the fully spatially coherent pump beam^35^. We have recently demonstrated the effects that the fully spatially coherent pump beam undergoes when passed through various strengths of atmospheric turbulence^36^. In this study we showed that the detection scheme of the single photon source is influenced by the changes in the atmospheric turbulence due to the breakdown of the spatial modes. Here, we showed for the first time the partially spatially coherent source for a quantum system. The present study might be useful for the preparation of two-qubit states by considering the partial spatial coherence of the pump beam for the application of free-space quantum communication. The coherence properties of the pump beam can be engineered to produce maximally entangled states^37^. This infers that a PSC pump beam within an entangled single photon source is a promising pump source in the application of free-space quantum communication.
